# 5-Methyl-*N*-(1,3-thia­zol-2-yl)isoxazole-4-carboxamide

**DOI:** 10.1107/S1600536813012105

**Published:** 2013-05-11

**Authors:** De-Cai Wang, Xiang Sun, Peng Su, Ping-Kai Ou-Yang

**Affiliations:** a State Key Laboratory of Materials-Oriented Chemical Engineering, School of Pharmaceutical Sciences, Nanjing University of Technology, Xinmofan Road No.5 Nanjing, Nanjing 210009, People’s Republic of China

## Abstract

In the title compound, C_8_H_7_N_3_O_2_S, the dihedral angle between the thia­zol and isoxazole rings is 34.08 (13)°. In the crystal, the mol­ecules are linked by pairs of N—H⋯N hydrogen bonds, forming inversion dimers, and C—H⋯O inter­actions, resulting in chains along the *b*-axis direction.

## Related literature

For background to isoxazole-containing drugs, see: Shaw *et al.* (2011 ▶); Schattenkirchner (2000 ▶); Huang *et al.* (2003 ▶). For the crystal structure of a related compound, see: Wang *et al.* (2011 ▶).
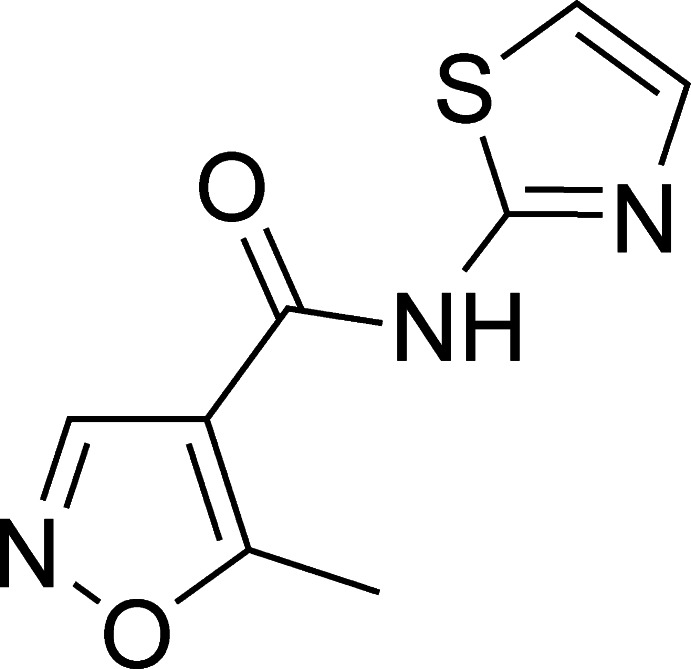


## Experimental

### 

#### Crystal data

C_8_H_7_N_3_O_2_S
*M* *_r_* = 209.23
Monoclinic, 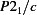

*a* = 8.8460 (18) Å
*b* = 10.742 (2) Å
*c* = 10.024 (2) Å
β = 107.27 (3)°
*V* = 909.6 (3) Å^3^
*Z* = 4
Mo *K*α radiation
μ = 0.33 mm^−1^
*T* = 293 K
0.30 × 0.20 × 0.10 mm

#### Data collection

Enraf–Nonius CAD-4 diffractometer
Absorption correction: ψ scan (North *et al.*, 1968 ▶) *T*_min_ = 0.907, *T*_max_ = 0.968
3462 measured reflections
1676 independent reflections
1298 reflections with *I* > 2σ(*I*)
*R*_int_ = 0.060
3 standard reflections every 200 reflections intensity decay: 1%

#### Refinement

*R*[*F* ^2^ > 2σ(*F* ^2^)] = 0.051
*wR*(*F* ^2^) = 0.152
*S* = 1.00
1676 reflections
128 parameters
H-atom parameters constrained
Δρ_max_ = 0.38 e Å^−3^
Δρ_min_ = −0.34 e Å^−3^

### 

Data collection: *CAD-4 EXPRESS* (Enraf–Nonius,1994) ▶; cell refinement: *CAD-4 EXPRESS*; data reduction: *XCAD4* (Harms & Wocadlo,1995 ▶); program(s) used to solve structure: *SHELXS97* (Sheldrick, 2008 ▶); program(s) used to refine structure: *SHELXL97* (Sheldrick, 2008 ▶); molecular graphics: *SHELXTL* (Sheldrick, 2008 ▶); software used to prepare material for publication: *PLATON* (Spek, 2009 ▶).

## Supplementary Material

Crystal structure: contains datablock(s) I, New_Global_Publ_Block. DOI: 10.1107/S1600536813012105/pv2631sup1.cif

Structure factors: contains datablock(s) I. DOI: 10.1107/S1600536813012105/pv2631Isup2.hkl

Supplementary material file. DOI: 10.1107/S1600536813012105/pv2631Isup3.cml

Additional supplementary materials:  crystallographic information; 3D view; checkCIF report

## Figures and Tables

**Table 1:** Hydrogen-bond geometry (Å, °)

*D*—H⋯*A*	*D*—H	H⋯*A*	*D*⋯*A*	*D*—H⋯*A*
N2—H2*A*⋯N1^i^	0.86	2.14	2.970 (3)	162
C6—H6*A*⋯O1^ii^	0.93	2.36	3.287 (4)	171

Symmetry codes: (i) 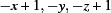; (ii) 
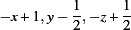
.

**Fig. 1.**
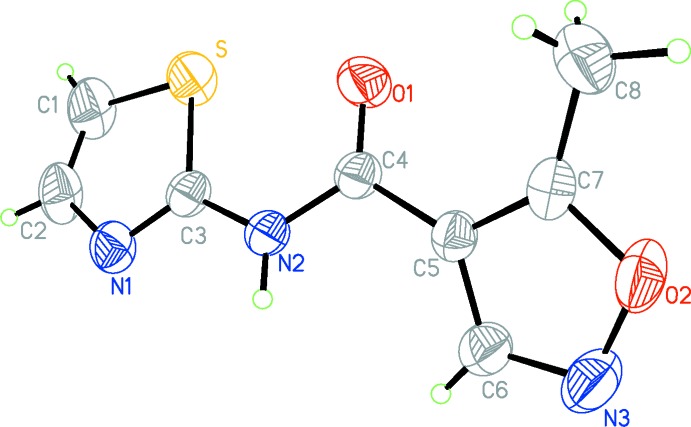
The molecular structure of the title compound with the atom numbering scheme. Displacement ellipsoids are drawn at the 35% probability level. H atoms are presented as small spheres of arbitrary radius.

**Fig. 2.**
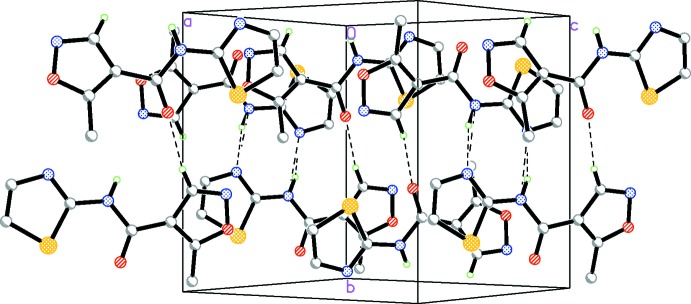
A view of the N—H···N and C—H···O hydrogen bonds (dotted lines) in the crystal structure of the title compound. H atoms non-participating in hydrogen-bonding were omitted for clarity.

## supplementary crystallographic information

### Comment

Leflunomide is one of the most effective isoxazole-containing disease-modifying drugs for treating rheumatoid arthritis (Shaw *et al.*, 2011; Schattenkirchner, 2000). Many leflunomide analogs have been synthesized which exhibit potent immunomodulating effect (Huang *et al.*, 2003). In our previous work, some anolog has been sucessfully sythesized (Wang *et al.*, 2011). A new leflunomide analog, *N*-5-methyl-*N*-(thiazol-2-yl)isoxazole-4-carboxamide, was synthesized in our laboratory as a novel and potent immunomodulating drug. In this paper we report its crystal structure.

The bond distances and angles in the title compound (Fig. 1) agree very well with the corresponding bond distances and angles reported in a closely related compound (Wang *et al.*, 2011). The dihedral angle between the C1/C2/N1/C3/S thiazol ring and the C5/C6/N3/O2/C7 isoxazole ring is 34.08 (13) °. In the crystal, the molecules are linked by N—H···N hydrogen bonds forming diamers about inversion centers and C—H···O hydrogen bonding interactions resulting in chains lying along the *b*-axis (Tab. 1 & Fig. 2).

### Experimental

A solution of 5-methylisoxazole-4-carboxylic acid chloride (7.3 g, 0.05 mol) in acetonitrile (20 ml) was added dropwise, while stirring, to thiazol-2-amine (12.9 g, 0.10 mol) dissolved in acetonitrile (150 ml), at room temperature. After stirring for 40 more minutes, the precipitated 5-methyl-*N*-(thiazol-2-yl)isoxazole-4-carboxamide was filtered off and washed with 100 ml portions of acetonitrile, and the combined filtrates were concentrated under reduced pressure yielded the title compouind as yellow crytalline product(Yield: 8.2 g; 60%). Crystals suitable for X-ray diffraction were obtained by slow evaporation of toluene solution.

### Refinement

All H atoms were placed geometrically at the distances of 0.93–0.96 Å for C—H and 0.86 Å for N—H and included in the refinement in riding motion approximation with *U*_iso_(H) = 1.2 or 1.5*U*_eq_ of the carrier atom.

### Crystal data

**Table:**

C_8_H_7_N_3_O_2_S	*F*(000) = 432
*M**_r_* = 209.23	*D*_x_ = 1.528 Mg m^−^^3^
Monoclinic, *P*2_1_/*c*	Mo *K*α radiation, λ = 0.71073 Å
Hall symbol: -P 2ybc	Cell parameters from 25 reflections
*a* = 8.8460 (18) Å	θ = 10–13°
*b* = 10.742 (2) Å	µ = 0.33 mm^−^^1^
*c* = 10.024 (2) Å	*T* = 293 K
β = 107.27 (3)°	Block, yellow
*V* = 909.6 (3) Å^3^	0.30 × 0.20 × 0.10 mm
*Z* = 4	

### Data collection

**Table:**

Enraf–Nonius CAD-4 diffractometer	1298 reflections with *I* > 2σ(*I*)
Radiation source: fine-focus sealed tube	*R*_int_ = 0.060
Graphite monochromator	θ_max_ = 25.4°, θ_min_ = 2.4°
ω/2θ scans	*h* = 0→10
Absorption correction: ψ scan (North *et al.*, 1968)	*k* = −12→12
*T*_min_ = 0.907, *T*_max_ = 0.968	*l* = −12→11
3462 measured reflections	3 standard reflections every 200 reflections
1676 independent reflections	intensity decay: 1%

### Refinement

**Table:**

Refinement on *F*^2^	Secondary atom site location: difference Fourier map
Least-squares matrix: full	Hydrogen site location: inferred from neighbouring sites
*R*[*F*^2^ > 2σ(*F*^2^)] = 0.051	H-atom parameters constrained
*wR*(*F*^2^) = 0.152	*w* = 1/[σ^2^(*F*_o_^2^) + (0.1*P*)^2^ + 0.180*P*] where *P* = (*F*_o_^2^ + 2*F*_c_^2^)/3
*S* = 1.00	(Δ/σ)_max_ < 0.001
1676 reflections	Δρ_max_ = 0.38 e Å^−^^3^
128 parameters	Δρ_min_ = −0.34 e Å^−^^3^
0 restraints	Extinction correction: *SHELXL*, Fc^*^=kFc[1+0.001xFc^2^λ^3^/sin(2θ)]^-1/4^
Primary atom site location: structure-invariant direct methods	Extinction coefficient: 0.021 (5)

### Special details

**Table:**

Geometry. All e.s.d.'s (except the e.s.d. in the dihedral angle between two l.s. planes) are estimated using the full covariance matrix. The cell e.s.d.'s are taken into account individually in the estimation of e.s.d.'s in distances, angles and torsion angles; correlations between e.s.d.'s in cell parameters are only used when they are defined by crystal symmetry. An approximate (isotropic) treatment of cell e.s.d.'s is used for estimating e.s.d.'s involving l.s. planes.
Refinement. Refinement of *F*^2^ against ALL reflections. The weighted *R*-factor *wR* and goodness of fit *S* are based on *F*^2^, conventional *R*-factors *R* are based on *F*, with *F* set to zero for negative *F*^2^. The threshold expression of *F*^2^ > σ(*F*^2^) is used only for calculating *R*-factors(gt) *etc*. and is not relevant to the choice of reflections for refinement. *R*-factors based on *F*^2^ are statistically about twice as large as those based on *F*, and *R*- factors based on ALL data will be even larger.

### Fractional atomic coordinates and isotropic or equivalent isotropic displacement parameters (Å^2^)

**Table:**

	*x*	*y*	*z*	*U*_iso_*/*U*_eq_	
S	0.27245 (9)	0.30807 (7)	0.46506 (9)	0.0578 (3)	
O1	0.5024 (2)	0.36916 (17)	0.3546 (2)	0.0517 (6)	
N1	0.3255 (3)	0.0745 (2)	0.5154 (2)	0.0463 (6)	
C1	0.1512 (4)	0.2266 (3)	0.5378 (4)	0.0629 (9)	
H1A	0.0654	0.2605	0.5607	0.075*	
O2	0.8730 (2)	0.2660 (2)	0.1941 (2)	0.0532 (6)	
N2	0.5011 (2)	0.16438 (19)	0.4088 (2)	0.0390 (5)	
H2A	0.5475	0.0938	0.4106	0.047*	
C2	0.1958 (3)	0.1078 (3)	0.5573 (3)	0.0558 (8)	
H2B	0.1426	0.0507	0.5969	0.067*	
N3	0.8148 (3)	0.1439 (3)	0.1552 (3)	0.0569 (7)	
C3	0.3761 (3)	0.1719 (2)	0.4640 (3)	0.0371 (6)	
C4	0.5547 (3)	0.2644 (2)	0.3512 (3)	0.0358 (5)	
C5	0.6762 (3)	0.2373 (2)	0.2825 (2)	0.0349 (5)	
C6	0.6993 (3)	0.1306 (3)	0.2088 (3)	0.0459 (6)	
H6A	0.6384	0.0587	0.1998	0.055*	
C7	0.7873 (3)	0.3192 (2)	0.2679 (3)	0.0395 (6)	
C8	0.8309 (4)	0.4479 (3)	0.3178 (3)	0.0546 (7)	
H8A	0.9169	0.4761	0.2855	0.082*	
H8B	0.8626	0.4493	0.4180	0.082*	
H8C	0.7413	0.5018	0.2821	0.082*	

### Atomic displacement parameters (Å^2^)

**Table:**

	*U*^11^	*U*^22^	*U*^33^	*U*^12^	*U*^13^	*U*^23^
S	0.0540 (5)	0.0548 (5)	0.0796 (6)	0.0179 (3)	0.0427 (4)	0.0136 (4)
O1	0.0510 (12)	0.0391 (10)	0.0740 (14)	0.0077 (8)	0.0325 (10)	0.0069 (9)
N1	0.0408 (12)	0.0511 (13)	0.0544 (13)	0.0009 (10)	0.0255 (10)	0.0066 (11)
C1	0.0458 (17)	0.081 (2)	0.076 (2)	0.0194 (16)	0.0384 (16)	0.0196 (18)
O2	0.0447 (11)	0.0687 (13)	0.0561 (12)	−0.0065 (9)	0.0302 (9)	−0.0037 (10)
N2	0.0395 (11)	0.0359 (10)	0.0499 (12)	0.0032 (9)	0.0260 (10)	0.0014 (9)
C2	0.0403 (15)	0.076 (2)	0.0595 (17)	0.0020 (14)	0.0282 (13)	0.0169 (15)
N3	0.0598 (15)	0.0630 (15)	0.0570 (15)	0.0005 (13)	0.0312 (12)	−0.0131 (12)
C3	0.0320 (12)	0.0434 (14)	0.0378 (13)	0.0024 (10)	0.0133 (10)	−0.0014 (10)
C4	0.0314 (12)	0.0372 (12)	0.0410 (13)	0.0014 (10)	0.0144 (10)	−0.0008 (10)
C5	0.0329 (12)	0.0382 (13)	0.0356 (12)	0.0004 (10)	0.0132 (10)	0.0014 (10)
C6	0.0498 (15)	0.0436 (14)	0.0482 (15)	−0.0031 (12)	0.0207 (12)	−0.0060 (12)
C7	0.0358 (13)	0.0501 (15)	0.0351 (13)	0.0006 (11)	0.0143 (11)	0.0039 (10)
C8	0.0531 (17)	0.0512 (16)	0.0616 (18)	−0.0123 (13)	0.0203 (14)	0.0034 (14)

### Geometric parameters (Å, º)

**Table:**

S—C1	1.707 (3)	N2—H2A	0.8600
S—C3	1.729 (2)	C2—H2B	0.9300
O1—C4	1.222 (3)	N3—C6	1.296 (4)
N1—C3	1.303 (3)	C4—C5	1.468 (3)
N1—C2	1.381 (3)	C5—C7	1.358 (4)
C1—C2	1.332 (5)	C5—C6	1.411 (4)
C1—H1A	0.9300	C6—H6A	0.9300
O2—C7	1.334 (3)	C7—C8	1.483 (4)
O2—N3	1.420 (3)	C8—H8A	0.9600
N2—C4	1.369 (3)	C8—H8B	0.9600
N2—C3	1.377 (3)	C8—H8C	0.9600
			
C1—S—C3	88.31 (14)	O1—C4—C5	122.1 (2)
C3—N1—C2	109.1 (2)	N2—C4—C5	115.9 (2)
C2—C1—S	111.0 (2)	C7—C5—C6	104.4 (2)
C2—C1—H1A	124.5	C7—C5—C4	125.2 (2)
S—C1—H1A	124.5	C6—C5—C4	130.3 (2)
C7—O2—N3	109.2 (2)	N3—C6—C5	112.4 (3)
C4—N2—C3	122.9 (2)	N3—C6—H6A	123.8
C4—N2—H2A	118.5	C5—C6—H6A	123.8
C3—N2—H2A	118.5	O2—C7—C5	109.3 (2)
C1—C2—N1	116.1 (3)	O2—C7—C8	117.0 (2)
C1—C2—H2B	122.0	C5—C7—C8	133.7 (3)
N1—C2—H2B	122.0	C7—C8—H8A	109.5
C6—N3—O2	104.7 (2)	C7—C8—H8B	109.5
N1—C3—N2	121.6 (2)	H8A—C8—H8B	109.5
N1—C3—S	115.55 (19)	C7—C8—H8C	109.5
N2—C3—S	122.86 (19)	H8A—C8—H8C	109.5
O1—C4—N2	122.0 (2)	H8B—C8—H8C	109.5
			
C3—S—C1—C2	0.8 (3)	N2—C4—C5—C7	−152.8 (2)
S—C1—C2—N1	−0.6 (4)	O1—C4—C5—C6	−145.8 (3)
C3—N1—C2—C1	−0.1 (4)	N2—C4—C5—C6	32.8 (4)
C7—O2—N3—C6	−0.6 (3)	O2—N3—C6—C5	−0.1 (3)
C2—N1—C3—N2	−177.4 (2)	C7—C5—C6—N3	0.8 (3)
C2—N1—C3—S	0.7 (3)	C4—C5—C6—N3	176.1 (3)
C4—N2—C3—N1	178.5 (2)	N3—O2—C7—C5	1.2 (3)
C4—N2—C3—S	0.6 (3)	N3—O2—C7—C8	−179.9 (2)
C1—S—C3—N1	−0.9 (2)	C6—C5—C7—O2	−1.2 (3)
C1—S—C3—N2	177.2 (2)	C4—C5—C7—O2	−176.7 (2)
C3—N2—C4—O1	5.7 (4)	C6—C5—C7—C8	−179.8 (3)
C3—N2—C4—C5	−173.0 (2)	C4—C5—C7—C8	4.6 (5)
O1—C4—C5—C7	28.5 (4)		

### Hydrogen-bond geometry (Å, º)

**Table:**

*D*—H···*A*	*D*—H	H···*A*	*D*···*A*	*D*—H···*A*
N2—H2*A*···N1^i^	0.86	2.14	2.970 (3)	162
C6—H6*A*···O1^ii^	0.93	2.36	3.287 (4)	171

Symmetry codes: (i) −*x*+1, −*y*, −*z*+1; (ii) −*x*+1, *y*−1/2, −*z*+1/2.
